# Impact of current and previous sperm findings on outcomes of intrauterine insemination

**DOI:** 10.1002/rmb2.12574

**Published:** 2024-04-08

**Authors:** Maki Taniguchi, Haruhiko Kanasaki, Aki Oride, Hiroe Okada, Kayo Imamura, Satoru Kyo

**Affiliations:** ^1^ Department of Obstetrics and Gynecology Shimane University Faculty of Medicine Izumo Japan; ^2^ Department of Obstetrics and Gynecology Unnan City Hospital Unnan Japan

**Keywords:** infertility, IUI, sperm findings

## Abstract

**Purpose:**

To examine the association between semen characteristics and outcomes of intrauterine insemination (IUI).

**Methods:**

This retrospective analysis examined 1380 IUI procedures involving 421 couples. The association of clinical pregnancy with pre‐ and post‐wash sperm characteristics was assessed.

**Results:**

Pre‐ and post‐wash sperm characteristics did not differ between IUI cycles that resulted in pregnancy and those that did not. When the motility of pre‐wash sperm was below the normal range (<42%) established by the World Health Organization (WHO), the pregnancy rate was significantly lower. In the IUI cycles when post‐wash sperm motility was below the WHO standard, pregnancy was not achieved. The frequency of improvement in post‐wash sperm motility in repeated IUI cycles appeared to correlate with the success of future IUI cycles. At the fourth IUI cycle, pregnancy was not achieved unless the post‐wash sperm motility was normal in at least two of three attempts. When post‐wash sperm concentration was below the normal range, the woman's age did not affect the IUI outcomes.

**Conclusions:**

Sperm motility above the lower limit of the WHO criteria in post‐wash semen samples is an important factor in IUI outcomes.

## 1 INTRODUCTION

Infertility affects millions of people of reproductive age worldwide and is considered a public health issue by World Health Organization.^1^ Among the various infertility treatments, intrauterine insemination (IUI) is widely recommended as the first‐line treatment for couples with subfertility when the woman has at least one normal patent fallopian tube and the man has normal or only mildly abnormal sperm parameters. IUI is noninvasive, easy to perform, and more affordable than other treatments for infertility.^2^, ^3^ Patients are generally compliant because little monitoring is necessary, and the risk for complications such as ovarian hyperstimulation syndrome is low.^4^ However, IUI procedures result in relatively low rates of pregnancy and live birth, and the cumulative pregnancy rate is reported to reach a plateau at 5 or 6 treatment cycles.^5^, ^6^, ^7^, ^8^ Several prognostic factors have been linked to IUI outcomes, including the type of ovarian stimulation, age of the female patient, duration of infertility, endometrial thickness, number of developing follicles, parameters of the sperm used in insemination, body mass index, and smoking.^9^ However, the predictive values of these parameters remain controversial.^10^ A recent study by Huang et al. compared factors influencing the outcomes of IUI between the pregnant group and the non‐pregnant group in order to elucidate any correlations and reported statistically significant differences in female anti‐mullerian hormone, endometrial thickness, and duration of ovarian stimulation.^11^

Although IUI treatment with or without ovarian stimulation is offered as a step‐up treatment to couples with subfertility, given the many variables potentially impacting the success rate, the role of IUI as an initial step for assisted reproductive technology (ART) remains controversial. In addition, IUI is considered a poor substitute for in vitro fertilization–embryo transfer (IVF‐ET), and the 2013 National Institute for Health and Care Excellence guidelines recommend that infertility patients with failed expectant management for up to 2 years should proceed directly to IVF.^12^ In addition, a previous multicenter randomized study reported that IUI with ovarian stimulation is non‐inferior compared with IVF and has a low multiple birth rate.^13^

Concerning the costs of infertility treatment, IUI is reported to be the most cost‐effective strategy and improves the live birth rate compared with expectant management for cases of mild male‐factor and unexplained infertility.^10^, ^14^ Therefore, IUI should be applied in appropriate cases of infertility prior to IVF treatment; however, it remains unclear how many IUI cycles should be attempted or in which cases multiple IUI attempts should be avoided and instead switched to a different strategy. In this study, we focused on the sperm parameters of successful cases of IUI treatment, including the pre‐ and post‐wash sperm findings when pregnancy was achieved by IUI; the change in post‐wash semen findings in cases where pregnancy was achieved; the relationship between the frequency of improvement in sperm findings after washing and the future IUI outcome; the semen findings in previous unsuccessful IUI cycles for pregnant cases; and the effect of aging on changes in sperm findings. In this study, we evaluated the pre‐ and post‐wash semen parameters at the time of IUI and investigated the association of these parameters with the success of IUI.

## 2 MATERIALS AND METHODS

### 2.1 Patients and study design

This is a retrospective observation study of all IUI procedures performed between January 2019 and December 2021 at Shimane University Hospital. Infertility was diagnosed in all couples in whom pregnancy was not achieved with 12 months or more of regular unprotected sexual intercourse. The main clinical outcome measure was clinical pregnancy rate per IUI cycle. The mean age of female patients was 35.8 ± 5.5 years (range, 21–51 years) and that of the male partners was 36.5 ± 6.2 years (range, 21–56 years). All insemination procedures performed for women over the age of 43 years at the time of insemination were included in the analysis, while canceled cycles were excluded. Follicular monitoring was performed in female patients until ovulation. Tubal patency was evaluated in all female patients, and those who had at least one unobstructed fallopian tube were included in the study. Sperm quality parameters were assessed before insemination, and cases with normal semen parameters or mild oligospermia were included in the study. This study was approved by the ethics committee of Shimane University Hospital.

### 2.2 Sperm preparation

On the day of the IUI procedure, semen samples were collected by masturbation into a sterile cup. Abstinence was not required prior to the commencement of IUI. After the semen was liquefied at room temperature, a basic wash and density‐gradient centrifugation were performed. The concentrated pellet was reconstituted to a volume of 0.3 mL with tubal media. Total volume, concentration, total number of sperm, motility rate, and total number of motile sperm in the pre‐ and post‐processing semen samples were determined prior to commencement of the IUI procedure. The total motile sperm count was obtained by multiplying the total sperm count by the percentage of motility. Sperm morphology was rated according to the World Health Organization (WHO) criteria.^1^ IUI cycles with donor sperm were excluded from the present study.

### 2.3 Intrauterine insemination

We included cases of IUI completed in unstimulated natural cycles, those with oral administration of 50–150 mg/day clomiphene citrate (Clomid tablets; Fuji Pharma Co., Ltd., Tokyo, Japan), and/or those with gonadotropin‐stimulated cycles. If the leading follicles were greater than 18 mm in diameter, the IUI procedures were planned and performed according to the couple's schedule. Muscular injection of 5000 IU human menopausal gonadotropin (Fuji Pharma Co., Ltd., Tokyo, Japan) or subcutaneous injection of choriogonadotropin alfa (Ovidrel Syringe; Merck Biopharma Co., Ltd., Tokyo, Japan) was administered 24 h prior to or on the day of the IUI procedure. The washed motile sperm (0.3 mL) were loaded into a soft catheter (Cook Medical Japan G.K., Tokyo, Japan) for insemination. Daily treatment with progesterone was not administered to the patients after the IUI procedure, but additional subcutaneous injection of 3000 IU human menopausal gonadotropin was given once at the discretion of doctor for some cases who were able to come to the hospital. Clinical pregnancy was confirmed by the presence of a gestational sac on ultrasonography 4–5 weeks after IUI.

### 2.4 Statistical analysis

Results are expressed as the mean ± standard deviation or percentage. Categorical data were compared using the Mann–Whitney *U* test, the Wilcoxon signed‐rank test, or the chi‐squared test according to the variables. Statistical significance was set at *p* < 0.05. Correlations between two metric variables were determined by Pearson's correlation coefficient. All analyses were performed using IBM SPSS statistics 26 (IBM Japan, Tokyo, Japan).

## 3 RESULTS

### 3.1 Background of pregnant and non‐pregnant couples

**FIGURE 1:**
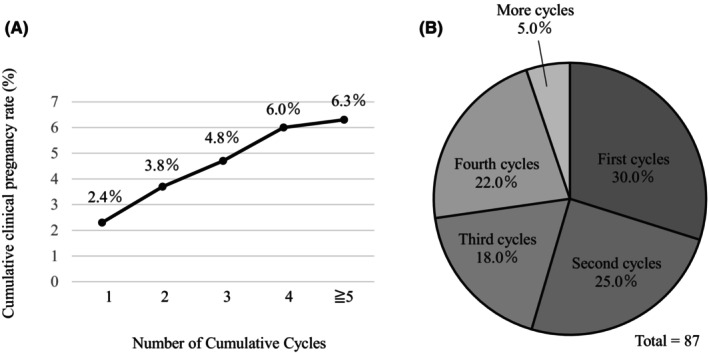
IUI cycles and pregnancy outcomes. (A) Relation between number of IUI cycles and the cumulative clinical pregnancy rate. (B) Cycle distribution in the success of IUI.

During the study period, 421 couples underwent a total of 1380 IUI cycles, using the partner's sperm. The mean age of female patients was 35.8 ± 5.5 years (range, 21–51 years) and that of the male partners was 36.5 ± 6.2 years (range, 21–56 years). Among the 1380 IUI procedures, clinical pregnancy was achieved in 87 IUI cycles of 81 women, giving pregnancy rates of 19.2% per patient and 6.3% per IUI cycle (Table 1). Among all IUI cycles, 2.4% of the couples achieved clinical pregnancy in the first cycle, and the cumulative pregnancy rate increased gradually with the number of IUI cycles (Figure 1A). Of the couples that achieved pregnancy, 30.0% did so during the first IUI cycle, the distribution of which is shown in Figure 1B. The mean age of women at the time of the IUI procedure resulting in pregnancy was 33.6 ± 4.3 years (range, 22–41 years), which was significantly lower compared with those who did not achieve pregnancy during their IUI cycles (36.0 ± 5.6 years). Similarly, the mean age of the male partner was significantly lower in couples who achieved pregnancy compared with those who did not (34.6 ± 5.5 vs. 36.6 ± 6.3 years) (Table 1).

**TABLE 1:** Participant characteristics.

	Total	Pregnant cases	Non‐pregnant cases	*p* ^a^
Number of couples	421	81 (19.2%)	353 (83.8%)	
Number of IUI cycles	1380	87 (6.3%)	1293 (93.7%)	
Woman's age (years) (mean ± SD)	35.8 ± 5.5	33.6 ± 4.4	36.0 ± 5.6	0.000
Range	21–51	22–41	21–51	
Partner's age (years) (mean ± SD)	36.5 ± 6.2	34.6 ± 5.5	36.6 ± 6.3	0.005
Range	21–56	22–48	21–56	

Abbreviation: SD, standard deviation.

^a^ Mann–Whitney *U* test.

### 3.2 Pre‐wash and post‐wash semen findings in pregnant and non‐pregnant couples

**FIGURE 2:**
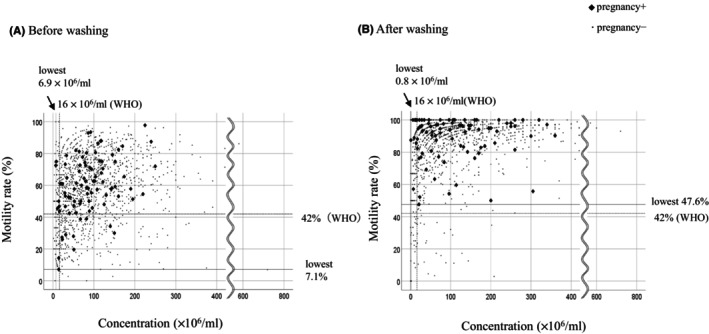
Sperm concentration and motility in pre‐ and post‐wash semen samples and the outcome of IUI. Pre‐wash (A) and post‐wash (B) sperm concentration (*x*‐axis) and motility (*y*‐axis) are plotted for each IUI cycle. IUI cycles that did not result in pregnancy are indicated by small dots, and those that resulted in pregnancy were indicated by black diamonds. The lower limits of the WHO standards for sperm concentration and motility are indicated by dashed lines. The lowest sperm concentration and motility rate that resulted in pregnancy in this study were also indicated by dashed lines.

Pre‐ and post‐wash semen findings were compared between IUI cycles in which pregnancy was achieved and those in which it was not (Table 2). Pre‐wash sperm volume, concentration, total number of sperm, motility rate, and total number of motile sperm did not significantly differ between IUI cycles that resulted in pregnancy and those that did not. Similarly, the post‐wash sperm parameters did not significantly differ between IUI cycles that resulted in pregnancy and those that did not. To determine the relation between pre‐ and post‐wash sperm parameters and the outcome of IUI, the concentration and motility rate of semen samples used in the IUI procedures and the IUI outcomes were plotted, and the cycles in which pregnancy was achieved were visualized (Figure 2). When focusing on the pre‐wash semen samples and IUI outcomes, the majority of pregnancies were achieved when semen motility was above 42% and semen concentration was above 16 × 10^6^/ml, both of which were above the lower limits of the WHO standards. However, in some cases pregnancy was achieved upon IUI despite pre‐wash sperm motility, concentration, or both being below the WHO standards (Figure 2A). Among the cases that achieved pregnancy, the lowest pre‐wash sperm concentration was 6.9 × 10^6^ and the worst motility rate was 7.1%. In Table 3, the couples were divided into two groups according to whether the original semen findings were within or outside the normal ranges of the WHO standards in terms of volume, concentration, motility, or both concentration and motility. The pregnancy rates for each IUI procedure were determined and compared between the two groups. There was no significant difference in pregnancy rate between the groups when the original semen volume was either over 1.4 cc or less than 1.4 cc, the latter of which is the lower limit of the WHO standard (6.2% vs. 6.3%). There was also no significant difference even when the original semen concentration was over the lower limit of the WHO standard (16 × 10^6^/ml) or less than the limit (6.3% vs. 5.4%). When IUI was performed using semen with a pre‐wash sperm motility rate of less than 42%, the pregnancy rate was 3.7%, but when IUI was performed using semen with a normal motility of >42%, the pregnancy rate was significantly higher at 6.9%. However, there was no significant difference in pregnancy rates between the two groups when both the concentration and motility rate were either above or below the WHO standards (6.9% vs. 3.4%) (Table 3). When IUI outcomes were examined according to their post‐wash semen findings, the majority of pregnant cases used semen samples with normal findings in the IUI procedure, although there were some exceptions (Figure 2B). The pregnancy rate was significantly lower when the post‐wash sperm concentration was below 16 × 10^6^/mL compared with when it was above 16 × 10^6^/mL (6.8% vs. 3.2%, *p* = 0.042). The lowest post‐wash concentration in a case resulting in pregnancy was 0.8 × 10^6^/mL. There were no cases of pregnancy when post‐wash sperm motility was below 42%. The worst post‐wash sperm motility in a case resulting in pregnancy was 47.6%. Similarly, there were no cases of pregnancy when both concentration and motility were below the WHO standards in post‐wash semen samples (Figure 2B and Table 3).

**TABLE 2:** Pre‐ and post‐wash semen findings in couples who did and did not achieve pregnancy.

		Pre‐washing semen			Post‐washing semen		
		Pregnant	Non‐pregnant	*p* ^a^	Pregnant	Non‐pregnant	*p* ^a^
Volume	Mean ± SD (mL)	2.9 ± 1.7	2.6 ± 1.6	0.179	0.3	0.3	
	Range	(0.5–8.7)	(0.2–29)				
Concentration	Mean ± SD (×10^6^/mL)	88.0 ± 56.5	96.1 ± 77.9	0.949	112.5 ± 91.7	108.6 ± 103.2	0.313
	Range	(6.9–250)	(1.5–720)		(0.8–360)	(0.0–720)	
Total sperm count	Mean ± SD (×10^6^)	245.7 ± 217.0	250.2 ± 261.5	0.541	33.7 ± 27.5	32.5 ± 30.9	0.313
	Range	(13.2–1104)	(0.6–2520)		(0.2–108)	(0.0–216)	
Motility rate	Mean ± SD (%)	59.7 ± 18.5	57.8 ± 20.5	0.576	88.8 ± 13.2	86.0 ± 17.9	0.267
	Range	(7.1–97.8)	(0.0–100)		(47.6–100)	(0.0–100)	
Total motile sperm count	Mean ± SD (×10^6^)	156.4 ± 160.4	153.8 ± 176.7	0.392	30.3 ± 25.5	29.2 ± 29.0	0.250
	Range	(2–966)	(0.0–1551.8)		(0.2–99)	(0.0–200.9)	
Abnormal rate	Mean ± SD (%)	80.0 ± 16.3	80.6 ± 18.0	0.376	63.9 ± 23.8	64.4 ± 25.5	0.635
	Range	(25–100)	(22.4–100)		(7.5–96.2)	(0.0–100)	

Abbreviation: SD, standard deviation.

^a^ Mann–Whitney *U* test.

**TABLE 3:** Pre‐ and post‐wash semen findings and IUI outcomes.

		Pre‐wash semen			Post‐wash semen		
		Within WHO standard	Below WHO standard	*p* ^a^	Within WHO standard	Below WHO standard	*p* ^a^
Volume (mL)	No. of cases	1159	221		–	–	
	Mean ± SD	3.0 ± 1.6	0.9 ± 0.3				
	Range	1.4–29	0.2–1.3				
	No. of pregnancies (%)	73 (6.2)	14 (6.3)	0.984			
Concentration (×10^6^/mL)	No. of cases	1252	128	0.683	1163	217	0.042
	Mean ± SD	104.3 ± 75.3	10.1 ± 3.6		127.7 ± 101.0	7.9 ± 4.1	
	Range	16–720	1.5–15		16–720	0.0–15	
	No. of pregnancies (%)	80 (6.3)	7 (5.4)		80 (6.8)	7 (3.2)	
Motility rate (%)	No. of cases	1086	294	0.042	1329	51	0.059
	Mean ± SD	66.1 ± 13.8	27.8 ± 10.0		88.6 ± 12.6	22.9 ± 12.5	
	Range	42–100	0.0–41.9		42.3–100	0.0–40.7	
	No. of pregnancies (%)	76 (6.9)	11 (3.7)		87 (6.5)	0 (0)	
Both concentration and motility rate	No. of cases	1016	58	0.298	1138	26	0.161
Concentration (×10^6^/mL)	Mean ± SD	108.2 ± 73.4	9.8 ± 3.6		128.9 ± 101.5	7.3 ± 5.2	
	Range	16–580	1.5–15		16–720	0.0–15	
Motility rate (%)	Mean ± SD	66.4 ± 13.7	24.6 ± 10.9		89.3 ± 11.5	23.2 ± 13.2	
	Range	42–97.8	0.0–41.7		42.3–100	0.0–40	
	No. of pregnancies (%)	71 (6.9)	2 (3.4)		80 (7.0)	0 (0)	

Abbreviation: SD, standard deviation.

^a^ Chi‐squared test.

### 3.3 Changes in the semen findings and outcome of IUI

**FIGURE 3:**
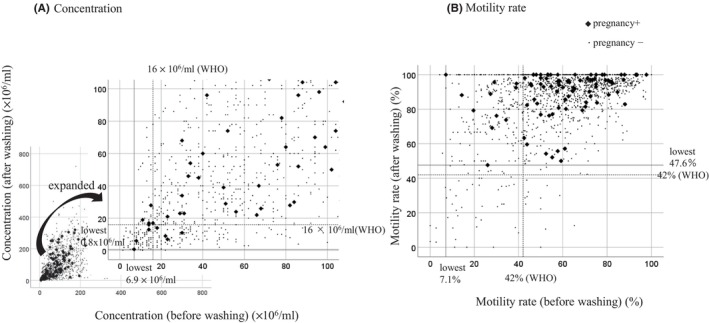
Changes in sperm concentration and motility rate in post‐wash semen samples. Changes in sperm concentration (A) and motility rate (B) are plotted for pre‐wash (*x*‐axis) and post‐wash (*y*‐axis) semen samples. IUI cycles that did not result in pregnancy are indicated by small dots, and those that resulted in pregnancy are indicated by black diamonds. The lower limits of the WHO standards for sperm concentration and motility are indicated by dashed lines. The lowest sperm concentration and motility rate that resulted in pregnancy in this study are also indicated by dashed lines.

Next, the changes in the sperm findings in the post‐wash semen samples and the IUI outcomes were investigated. The changes in sperm parameters between pre‐ and post‐wash sperm samples were plotted, and the cycles in which pregnancy was achieved were visualized. Figure 3A shows the changes in sperm concentration for the pre‐ and post‐wash samples. The extent to which sperm concentration improved varied according to the sample. When the original semen concentration was below the WHO standard (16 × 10^6^/mL) and could not be improved to above the normal range by washing, pregnancy was still achieved in some cases. Similarly, even when the sperm concentration in the original semen sample was above the WHO standard and it worsened by washing, pregnancy could still be achieved. However, in the majority of cases, pregnancy was achieved when the pre‐wash sperm concentration was normal or when the post‐wash concentration had improved to normal (Figure 3A). Changes in post‐wash sperm motility also varied among cases. In some cases, washing did not improve sperm motility that was below the WHO standard. Also, sperm motility that was above the WHO standard was exacerbated by washing in same cases. However, in all cases where pregnancy was achieved, the post‐wash sperm motility was above the WHO normal range of 42% (Figure 3B).

### 3.4 Previous sperm findings and outcome of IUI

To determine whether the frequency of improvement in sperm findings after washing was related to IUI outcomes, the rate of improvement in sperm parameters after washing and cases of pregnancy were examined among couples who underwent IUI three or four times. Sperm improvement was defined according to whether the parameters of the pre‐wash sperm improved after washing, regardless of whether it reached the WHO standards or not. As shown in Table 4, a total of 80 couples underwent IUI three times. Among them, the partner's sperm motility improved each time in 21 cases (26.2%), whereas in 15 cases (18.8%), post‐wash sperm concentration did not improve all three times. Sperm motility rate was more frequently improved after washing. Among the 80 cases who tried IUI a third time, the sperm motility rate improved all three times after washing in 65 cases (81.2%) and two of three times in 13 cases (16.3%); there were no cases in which the sperm motility rate improved after washing. There were no pregnancies in cases in which sperm concentration did not improve after all three washes, but there did not seem to be any relation between the frequency of improvement in sperm concentration and the pregnancy rate. However, the pregnancy rate was higher in cases in which sperm motility improved all three times compared with cases in which sperm motility improved two out of three times (13.7% vs. 2.5%). Similarly, among the cases that tried IUI four times, the frequency of improvement in post‐wash sperm motility, but not concentration, appeared to be correlated with the success of IUI (Table 4).

**TABLE 4:** Frequency in improvement of sperm concentration and motility rate in the current and previous post‐wash semen samples and IUI outcomes.

3rd attempt		Improved 3/3 times	Improved 2/3 times	Improved 1/3 times	Improved 0/3 times
Total 80 Pregnant 13		Concentration			
	No. of cycles (% of total cycles)	21 (26.2)	16 (20.0)	28 (35.0)	15 (18.8)
	No. of pregnancy (% of total cycles)	4 (5.0)	3 (3.8)	6 (7.5)	0 (0)
		Motility			
	No. of cycles (% of total cycles)	65 (81.2)	13 (16.3)	2 (2.5)	0 (0)
	No. of pregnancy (% of total cycles)	11 (13.7)	2 (2.5)	0 (0)	0 (0)

4th attempt		Improved 4/4 times	Improved 3/4 times	Improved 2/4 times	Improved 1/4 times	Improved 0/4 times
Total 74 Pregnant 14		Concentration				
	No. of cycles (% of total cycles)	13 (17.6)	13 (17.6)	24 (32.4)	17 (23.0)	7 (9.4)
	No. of pregnancy (% of total cycles)	1 (1.3)	3 (4.1)	3 (4.1)	6 (8.1)	1 (1.4)
		Motility				
	No. of cycles (% of total cycles)	62 (83.8)	11 (14.9)	1 (1.3)	0 (0)	0 (0)
	No. of pregnancy (% of total cycles)	12 (16.2)	2 (9.5)	0 (0)	0 (0)	0 (0)

To determine whether sperm findings in two or more previous unsuccessful IUI cycles could predict future IUI outcomes, we examined the previous post‐wash semen findings in 13 women who became pregnant on their third IUI attempt as well as those of 14 women who became pregnant on their fourth IUI attempt (Table 5). In 11 of the 13 women who became pregnant on their third attempt, the post‐wash sperm concentration was normal (>16 × 10^6^) in both of the previous two unsuccessful cycles. In the other 2 women, the sperm concentration improved to normal in one of the two unsuccessful attempts. There were no cases of pregnancy in which the sperm concentration did not normalize after washing. In the 13 women who achieved pregnancy on their third IUI attempt, the post‐wash sperm motility was above 42% in both of the previous two unsuccessful cycles. In the 14 women who became pregnant on their fourth IUI attempt, 6 (42.9%) had used semen with a sperm concentration above the WHO standard in their previous three unsuccessful cycles. In 4 and 3 pregnant women who became pregnant on their fourth attempt, the previous post‐wash sperm concentration was normalized twice and once, respectively, in their previous three attempts. There was one case of pregnancy in which the post‐wash sperm concentration did not normalize in any of the previous three cycles. Meanwhile, 13 of the 14 women who became pregnant on their fourth IUI attempt (92.9%) had normal motility in the previous three unsuccessful IUI cycles, while the remaining woman had normal motility sperm in two of the three unsuccessful cycles.

**TABLE 5:** Normalization of sperm concentration and motility rate in the previous post‐wash semen samples and IUI outcomes.

Pregnancy achieved on 3rd attempt			Normalized 2/2 times	Normalized 1/2 times	Normalized 0/2 times	
13 Cases	No. of pregnancies	Concentration	11	2	0	
		Motility	13	0	0	

Pregnancy achieved on 4th attempt			Normalized 3/3 times	Normalized 2/3 times	Normalized 1/3 times	Normalized 0/3 times
14 Cases	No. of pregnancies	Concentration	6	4	3	1
		Motility	13	1	0	0

### 3.5 Women's age and IUI outcome and partners' age and post‐wash sperm findings

**FIGURE 4:**
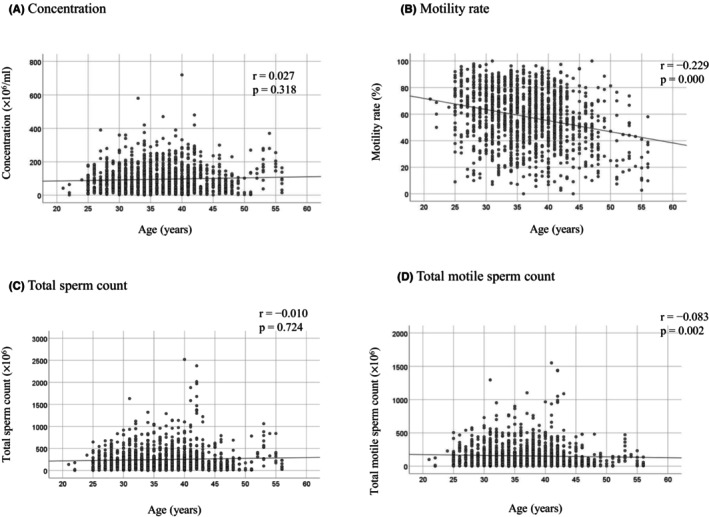
Relationship between age and semen parameters in the male partners of women who underwent IUI. Pre‐wash semen concentration (A), motility rate (B), total sperm count (C), and total motile sperm count (D) are plotted according to age. The correlation between the two metric variables was determined by Pearson's correlation coefficient.

To examine whether a woman's age might compensate for poor semen findings, the pregnancy rates of women under the age of 35 years and those over the age 35 years were compared in terms of normal and abnormal post‐wash sperm concentration. In all cases, pregnancy rates were significantly higher in women under the age of 35 years compared with those over the age 35 years (8.3% vs. 4.7%, *p* = 0.008). This trend was also observed when comparing IUI cycles in which post‐wash semen concentrations were normal (>16 × 10^6^/ml) (8.8% vs. 5.3%, *p* = 0.018). In contrast, pregnancy rates did not significantly differ between these two age groups when post‐wash sperm concentrations were below the normal range (4.9% vs. 2.2%, *p* = 0.271) (Table 6). The changes in pre‐wash sperm findings by partner's age were also examined. Sperm concentration (Figure 4A), total sperm count (Figure 4C), and total motile sperm counting (Figure 4D) were not correlated with partner's age; only the motility rate in pre‐wash sperm showed a slight negative correlation with the male partner's age (Figure 4B).

**TABLE 6:** Post‐wash sperm concentrations and IUI outcomes in women ≤35 years and >35 years of age.

		Age ≤35 years	Age >35 years	*p* ^a^
Total	No. of cycles	588	792	0.008
	Concentration (×10^6^/mL)	111.9 ± 96.7	106.6 ± 106.6	
	Range	0–720	0.1–600	
	Cases of pregnancy (%)	49 (8.3)	38 (4.7)	
Within WHO standard	No. of cycles	507	656	0.018
	Concentration (×10^6^/mL)	128.4 ± 94.1	127.0 ± 106.2	
	Range	16–720	16–600	
	Cases of pregnancy (%)	45 (8.8)	35 (5.3)	
Below WHO standard	No. of cycles	81	136	0.271
	Concentration (×10^6^/mL)	8.1 ± 4.1	7.8 ± 4.2	
	Range	0.0–15	0.1–15	
	Cases of pregnancy (%)	4 (4.9)	3 (2.2)	

^a^ Chi‐squared test.

## 4 DISCUSSION

IUI treatment has a central role in the treatment of infertile couples with ovulatory disorders or unexplained infertility. Ejaculatory disorder and mild‐to‐moderate male‐factor infertility are also eligible for IUI treatment prior to IVF.^15^ The success rate of IUI per cycle is generally low compared with IVF; nevertheless, clinical practice provides evidence that it is a worthwhile step for couples who are considering IVF treatment. Although the success of IUI is largely affected by the woman's age,^16^, ^17^ the success of IUI may also be influenced by the duration of infertility, the ovarian stimulation protocol, infertility etiology, number of treatment cycles, and timing of insemination.^6^, ^18^ In addition, recent evidence suggests that male‐factor infertility also contributes to IUI outcomes, and it has been reported that total motile sperm count is an important parameter in semen analysis.^19^ In the present study, we evaluated semen samples used in 1380 IUI cycles performed between January 2019 and December 2021 at Shimane University Hospital with the aim of determining whether semen findings are predictive of present and future IUI outcomes. Sperm parameters in pre‐wash semen samples were compared with those in post‐wash semen samples, and the relationship between pre‐ and post‐wash semen findings and IUI outcomes was examined. Furthermore, we investigated whether the semen findings used in previous unsuccessful IUI cycles could predict the success of future IUI cycles in order to obtain helpful information for determining whether to continue IUI or switch to ART. To accurately determine the IUI outcomes at our institution, and to analyze as many semen samples used in previous IUI cycles as possible, we targeted all women aged 21 to 51 who had undergone IUI treatment in our hospital.

A simple comparison of pre‐wash and post‐wash sperm findings between IUI cycles in which pregnancy was achieved and those in which it was not revealed no significant differences in any of the sperm parameters. The mean semen volume, sperm concentration, total sperm count, sperm motility, total motile sperm count, and abnormal rate in the pre‐wash or post‐wash semen samples were all similar between IUI cycles that resulted in pregnancy and those that did not. Similarly, the sperm parameters in the post‐wash semen samples did not differ significantly between IUI cycles that resulted in pregnancy and those that did not. Taken together, these results suggested that both pre‐wash and post‐wash sperm findings were mostly the same in IUI cycles that resulted in pregnancy and those that did not.

There were cases in which pregnancy was achieved even when the original semen parameters were below the WHO standards, that is, volume below 1.4 mL, concentration below 16 × 10^6^ sperm/mL, and motility below 42%. The minimal semen volume that resulted in pregnancy was 0.2 mL, the lowest concentration was 6.9 × 10^6^/mL, and the lowest motility was 7.1% in the pre‐wash sperm samples. Therefore, because it is possible to conceive by IUI even when the original semen findings are below the WHO standards, infertile couples should not be rushed to ART without first trying IUI, based solely on poor semen findings. When the outcomes of IUI procedures were examined in terms of whether the pre‐wash semen findings were within or outside of range of the WHO standards, the pregnancy rate did not significantly differ regardless of whether the quantity, concentration, or both concentration and motility did not satisfy the WHO standards. However, when IUI was performed using semen with a normal motility of >42%, the pregnancy rate was significantly higher compared with that in IUI cycles using semen with a motility below the WHO standard (<42%). We consider that the sperm quality necessary for successful IUI is lower than the WHO threshold values for normal sperm, as previously reported.^20^ However, we should note that the sperm motility in the original semen findings is more sensitive to the success of IUI. Although some reports have indicated that pre‐wash semen findings are not related to IUI outcomes,^11^, ^21^ others have suggested the importance of initial pre‐wash sperm motility in successful IUI.^22^

Generally, semen parameters are improved by washing, and the post‐wash semen is used for insemination. Although there was a slight association with motility rate, poor pre‐wash semen findings were not closely associated with the IUI outcomes: However, when post‐wash sperm findings of concentration and motility were below the WHO standards, pregnancy rates were significantly low compared with cases in post‐wash semen samples with parameters above the WHO standards. There were cases in which pregnancy was achieved even when the post‐wash sperm concentration was below the WHO standard (16 × 10^6^/mL), but the pregnancy rate was low compared with cases in which the post‐wash sperm concentration was above the WHO standard. However, there were no IUI cycles in which pregnancy was achieved when the motility rate of post‐wash sperm was below the WHO standard (<42%). Pasqualotto et al. reported that post‐wash sperm motility, but not pre‐wash motility, was predictive of successful IUI outcomes with a cut‐off value of 40%, which was the previous WHO standard issued in 2010.^23^ This is also supported by previous studies.^24^, ^25^ Our observations suggest that the same could be said based on the 2021 WHO standard; that is, when the post‐wash sperm motility is less than 42%, IUI is unlikely to succeed.

In this study, we also showed the changes in sperm parameters in semen samples used in individual IUI cycles. The changes in sperm concentration by sperm washing varied in each semen sample. Even when the original sperm concentration was below the WHO standard (16 × 10^6^/mL), it could be improved to normal concentration levels after washing. However, in some cases the normal sperm concentration worsened or the low sperm concentration remained low after washing. Because pregnancy was achieved in some cases even when the post‐wash sperm concentration was below the WHO standard and because successful IUI cycles did not always have the best sperm concentration compared with previous unsuccessful IUI cycles, it can be said that pre‐ and post‐wash sperm concentration is not a predictor of the success of IUI. In contrast, although the improvement in sperm motility by washing also varied in each case, all cases of pregnancy had a motility rate above 42%, which is the lower limit of the WHO standard. Pregnancy was not achieved when post‐wash sperm had a motility of less than 42%. In addition, our investigation of post‐wash sperm findings in sequential IUI cycles revealed that 11 of 13 couples (84.6%) who achieved pregnancy at their third IUI cycle had normal concentrations of post‐wash sperm in the previous two unsuccessful IUI cycles. In addition, all couples who achieved pregnancy on their third IUI cycle had normal (>42%) post‐wash sperm motility rates in the previous two unsuccessful IUI cycles. This information should be considered when deciding whether to perform another IUI cycle. Although post‐wash sperm motility was always above 42% when pregnancy was achieved by IUI, among the 14 couples who successfully achieved pregnancy at their fourth IUI cycle, 13 (92.8%) had normal motility in the previous three unsuccessful IUI cycles. The remaining couple who achieved pregnancy at their fourth IUI cycle had normal motility in two of the previous three unsuccessful IUI cycles. This is also valuable information to consider when deciding whether to attempt a fourth IUI cycle.

We retrospectively reviewed sperm findings in all semen samples used in IUI cycles in the study period. It is unclear whether semen quality is affected by aging. Loverira et al. reported that semen quality seems to be influenced by aging,^26^ while Tatsumi et al. reported that advanced paternal age does not adversely affect sperm parameters.^27^ In the present study, we showed that among the pre‐wash semen parameters, sperm motility was the only parameter that was negatively correlated with partner's age, albeit slightly. Total sperm count, concentration, and total motile sperm count were not correlated with partner's age. Women are less likely to conceive as age increases, and in cases of spontaneous conception, the partner's age is likely to be a factor. As observed in the cases of IUI that resulted in pregnancy in this study, sperm motility probably needs to be 42% or higher, even in cases of spontaneous conception. However, when undergoing IUI treatment, even if sperm motility is decreased due to the partner's age, it should not affect the outcome of IUI as long as the post‐wash sperm motility rate is over 42%. The outcome of IUI is reported to be dependent on the women's age,^28^ which is in line with the results of the present study. However, pregnancy rates did not significantly differ according to the women's age in IUI with sperm concentrations below the normal range of the WHO standard. These findings suggest that the higher oocyte quality in younger women does not compensate for low sperm concentrations in achieving pregnancy with IUI.

In this study, we focused on the sperm parameters of successful cases of IUI treatment. Pre‐ and post‐wash sperm characteristics did not differ between IUI cycles that resulted in pregnancy and those that did not. When the motility of pre‐wash sperm was below the normal range established by the WHO (i.e., <42%), the pregnancy rate was significantly lower. In the IUI cycles in which post‐wash sperm motility was below the WHO standard, pregnancy was not achieved. At the third IUI cycle, there were no cases of pregnancy unless the post‐wash sperm motilities used in the previous two IUI procedures were above 42%. At the fourth IUI cycle, pregnancy was not achieved unless the post‐wash sperm motility was normal in at least two of the three previous attempts. From these findings, sperm motility above the lower limit of the WHO criteria is thought to be an important factor in IUI outcomes. Although this study was retrospective in nature, the results provide additional insight into the indications for future IUI treatment.

## CONFLICT OF INTEREST STATEMENT

The authors declare no conflict of interest.

## ETHICS STATEMENT

The study design was reviewed and approved by the institutional review board of Shimane University Hospital. All procedures were conducted in accordance with the ethical standards of the responsible committee on human experimentation (institutional and national) and with the 1964 Declaration of Helsinki and its later amendments. Informed consent was obtained from all patients prior to being included in the study.

## References

[1] Ombelet W . WHO fact sheet on infertility gives hope to millions of infertilie couples world wide. Facts Views Vis Obgyn. 2020;12(4):249–251. 33575673 PMC7863696

[2] Allahbadia GN . Intrauterine insemination: fundamentals revisited. J Obstet Gynaecol India. 2017;67(6):385–392. 29162950 10.1007/s13224-017-1060-x PMC5676579

[3] Schlegel PN , Sigman M , Collura B , De Jonge CJ , Eisenberg ML , Lamb DJ , et al. Diagnosis and treatment of infertility in men: AUA/ASRM guideline part I. J Urol. 2021;205(1):36–43. 33295257 10.1097/JU.0000000000001521

[4] Ombelet W . The revival of intrauterine insemination: evidence‐based data have changed the picture. Facts Views Vis Obgyn. 2017;9(3):131–132. 29479397 PMC5819320

[5] Duran HE , Morshedi M , Kruger T , Oehninger S . Intrauterine insemination: a systematic review on determinants of success. Hum Reprod Update. 2002;8(4):373–384. 12206471 10.1093/humupd/8.4.373

[6] Nesbit CB , Blanchette‐Porter M , Esfandiari N . Ovulation induction and intrauterine insemination in women of advanced reproductive age: a systematic review of the literature. J Assist Reprod Genet. 2022;39(7):1445–1491. 35731321 10.1007/s10815-022-02551-8 PMC9365895

[7] Nuojua‐Huttunen S , Tomas C , Bloigu R , Tuomivaara L , Martikainen H . Intrauterine insemination treatment in subfertility: an analysis of factors affecting outcome. Hum Reprod. 1999;14(3):698–703. 10221698 10.1093/humrep/14.3.698

[8] Immediata V , Patrizio P , Parisen Toldin MR , Morenghi E , Ronchetti C , Cirillo F , et al. Twenty‐one year experience with intrauterine inseminations after controlled ovarian stimulation with gonadotropins: maternal age is the only prognostic factor for success. J Assist Reprod Genet. 2020;37(5):1195–1201. 32215826 10.1007/s10815-020-01752-3 PMC7244676

[9] Luco SM , Agbo C , Behr B , Dahan MH . The evaluation of pre and post processing semen analysis parameters at the time of intrauterine insemination in couples diagnosed with male factor infertility and pregnancy rates based on stimulation agent. A retrospective cohort study. Eur J Obstet Gynecol Reprod Biol. 2014;179:159–162. 24965998 10.1016/j.ejogrb.2014.05.003 PMC4144991

[10] Farquhar CM , Liu E , Armstrong S , Arroll N , Lensen S , Brown J . Intrauterine insemination with ovarian stimulation versus expectant management for unexplained infertility (TUI): a pragmatic, open‐label, randomised, controlled, two‐centre trial. Lancet. 2018;391(10119):441–450. 29174128 10.1016/S0140-6736(17)32406-6

[11] Huang X , Sun Q , Tang X , Li M , Zhou C , Cheng X , et al. Factors influencing the pregnancy outcome of intrauterine insemination and follow‐up treatment. J Hum Reprod Sci. 2023;16(1):42–49. 37305770 10.4103/jhrs.jhrs_130_22 PMC10256937

[12] O'Flynn N . Assessment and treatment for people with fertility problems: NICE guideline. Br J Gen Pract. 2014;64(618):50–51. 24567574 10.3399/bjgp14X676609 PMC3876144

[13] Bensdorp AJ , Tjon‐Kon‐Fat RI , Bossuyt PM , Koks CA , Oosterhuis GJ , Hoek A , et al. Prevention of multiple pregnancies in couples with unexplained or mild male subfertility: randomised controlled trial of in vitro fertilisation with single embryo transfer or in vitro fertilisation in modified natural cycle compared with intrauterine insemination with controlled ovarian hyperstimulation. BMJ. 2015;350:g7771. 25576320 10.1136/bmj.g7771 PMC4288434

[14] Tjon‐Kon‐Fat RI , Bensdorp AJ , Bossuyt PM , Koks C , Oosterhuis GJ , Hoek A , et al. Is IVF‐served two different ways‐more cost‐effective than IUI with controlled ovarian hyperstimulation? Hum Reprod. 2015;30(10):2331–2339. 26269539 10.1093/humrep/dev193

[15] Cohlen B , Bijkerk A , Van der Poel S , Ombelet W . IUI: review and systematic assessment of the evidence that supports global recommendations. Hum Reprod Update. 2018;24(3):300–319. 29452361 10.1093/humupd/dmx041

[16] Haebe J , Martin J , Tekepety F , Tummon I , Shepherd K . Success of intrauterine insemination in women aged 40–42 years. Fertil Steril. 2002;78(1):29–33. 12095486 10.1016/s0015-0282(02)03168-0

[17] Ruiter‐Ligeti J , Dahan MH , Steiner N , Volodarsky‐Perel A , Buckett W . Is intrauterine insemination a viable treatment option for women over 43 years old? An analysis by ovarian stimulation protocol and sperm source. J Assist Reprod Genet. 2020;37(12):3103–3107. 33107579 10.1007/s10815-020-01976-3 PMC7714793

[18] Merviel P , Heraud MH , Grenier N , Lourdel E , Sanguinet P , Copin H . Predictive factors for pregnancy after intrauterine insemination (IUI): an analysis of 1038 cycles and a review of the literature. Fertil Steril. 2010;93(1):79–88. 18996517 10.1016/j.fertnstert.2008.09.058

[19] Muthigi A , Jahandideh S , Bishop LA , Naeemi FK , Shipley SK , O'Brien JE , et al. Clarifying the relationship between total motile sperm counts and intrauterine insemination pregnancy rates. Fertil Steril. 2021;115(6):1454–1460. 33610321 10.1016/j.fertnstert.2021.01.014

[20] Dickey R , Sartor SM , Pryzak R . Efficacy of superovulation and intrauterine insemination in the treatment of infertility. N Engl J Med. 1999;341(2):128. 10.1056/NEJM199907083410214 10409029

[21] Madbouly K , Isa A , Habous M , Almannie R , Abu‐Rafea B , Binsaleh S . Postwash total motile sperm count: should it be included as a standard male infertility work up. Can J Urol. 2017;24(3):8847–8852. 28646941

[22] Jeong M , Kim SK , Kim H , Lee JR , Jee BC , Kim SH . Predictive value of sperm motility before and after preparation for the pregnancy outcomes of intrauterine insemination. Clin Exp Reprod Med. 2021;48(3):255–261. 34488289 10.5653/cerm.2021.04469 PMC8421663

[23] Pasqualotto EB , Daitch JA , Hendin BN , Falcone T , Thomas AJ Jr , Nelson DR , et al. Relationship of total motile sperm count and percentage motile sperm to successful pregnancy rates following intrauterine insemination. J Assist Reprod Genet. 1999;16(9):476–482. 10530401 10.1023/A:1020598916080 PMC3455631

[24] Atasever M , Kalem MN , Hatirnaz S , Hatirnaz E , Kalem Z , Kalaylioglu Z . Factors affecting clinical pregnancy rates after IUI for the treatment of unexplained infertility and mild male subfertility. J Turk Ger Gynecol Assoc. 2016;17(3):134–138. 27651720 10.5152/jtgga.2016.16056 PMC5019828

[25] Shulman A , Hauser R , Lipitz S , Frenkel Y , Dor J , Bider D , et al. Sperm motility is a major determinant of pregnancy outcome following intrauterine insemination. J Assist Reprod Genet. 1998;15(6):381–385. 9673883 10.1023/A:1022585000740 PMC3455014

[26] Oliveira JBA , Petersen CG , Mauri AL , Vagnini LD , Baruffi RLR , Franco JG Jr . The effects of age on sperm quality: an evaluation of 1,500 semen samples. JBRA Assist Reprod. 2014;18(2):34–41. 35761724 10.5935/1518-0557.20140002 PMC9236654

[27] Tatsumi T , Ishida E , Tatsumi K , Okada Y , Saito T , Kubota T , et al. Advanced paternal age alone does not adversely affect pregnancy or live‐birth rates or sperm parameters following intrauterine insemination. Reprod Med Biol. 2018;17(4):459–465. 30377400 10.1002/rmb2.12222 PMC6194307

[28] Youn JS , Cha SH , Park CW , Yang KM , Kim JY , Koong MK , et al. Predictive value of sperm motility characteristics assessed by computer‐assisted sperm analysis in intrauterine insemination with superovulation in couples with unexplained infertility. Clin Exp Reprod Med. 2011;38(1):47–52. 22384418 10.5653/cerm.2011.38.1.47 PMC3283051

